# Tamponade and massive pleural effusions secondary to peripherally inserted central catheter in neonates–A complication to be aware of

**DOI:** 10.3389/fcvm.2023.1092814

**Published:** 2023-02-17

**Authors:** Rana Zareef, Mariam Anka, Taha Hatab, Issam El Rassi, Khalid Yunis, Fadi Bitar, Mariam Arabi

**Affiliations:** ^1^Faculty of Medicine, American University of Beirut Medical Center, Beirut, Lebanon; ^2^Division of Pediatric Cardiology, Department of Pediatrics and Adolescent Medicine, American University of Beirut Medical Center, Beirut, Lebanon; ^3^Department of Surgery, American University of Beirut Medical Center, Beirut, Lebanon; ^4^Division of Neonatology, Department of Pediatrics and Adolescent Medicine, American University of Beirut, Beirut, Lebanon

**Keywords:** tamponade, pleural effusions, pericardial effusions, central catheter, neonates

## Abstract

**Background:**

Peripherally inserted central catheters (PICC) are frequently used in neonatal intensive care units (NICU) to assist premature and critically ill neonates. Massive pleural effusions, pericardial effusions, and cardiac tamponade secondary to PICC are extremely uncommon but have potentially fatal consequences.

**Objective:**

This study investigates the incidence of tamponade, large pleural, and pericardial effusions secondary to peripherally inserted central catheters in a neonatal intensive care unit at a tertiary care center over a 10-year period. It explores possible etiologies behind such complications and suggests preventative measures.

**Study design:**

Retrospective analysis of neonates who were admitted to the NICU at the AUBMC between January 2010 and January 2020, and who required insertion of PICC. Neonates who developed tamponade, large pleural, or pericardial effusions secondary to PICC insertion were investigated.

**Results:**

Four neonates developed significant life-threatening effusions. Urgent pericardiocentesis and chest tube placement were required in two and one patients, respectively. No fatalities were encountered.

**Conclusion:**

The abrupt onset of hemodynamic instability without an obvious cause in any neonate with PICC *in situ* should raise suspicion of pleural or pericardial effusions. Timely diagnosis through bedside ultrasound, and prompt aggressive intervention are critical.

## 1. Introduction

Over the last few decades, advancements in the field of neonatology have resulted in an increase in the overall survival of critically ill, extremely premature, and very low birthweight neonates. Maintaining long-term intravenous access to administer pharmacologic and nutritional support in this population is essential yet is often challenging (1). Therefore, a central line is typically used in neonates. The placement of peripherally inserted central catheters (PICC) has become a common practice in neonatal intensive care units (NICU). A neonatal PICC (nPICC), also known as a percutaneous indwelling central catheter, is a type of intravenous access that can be used for extended periods of time and is typically used for the long-term administration of total parenteral nutrition (TPN), blood products, and medications (2). Routinely, the PICC protrudes the skin at a peripheral location, reaches the superior vena cava, and resides inside for days or weeks (1). Neonatal catheters are typically inserted through a superficial vein, and are therefore called Epicutaneo-caval catheters (3). In general, neonatal PICC was first described in 1973 by Shaw as a technique for inserting a silicone catheter into the central veins of neonates (4). Since then, there has been significant improvement in the practice of insertion (5). Although PICCs are relatively easy and quick to obtain, they are not risk-free and have been associated with various complications such as occlusion, infection, thrombosis, breakage, and migration. Even life-threatening pleural and pericardial effusions have been documented (6).

The NICU at the American University of Beirut Medical Center (AUBMC) is an active referral center that admits more than 250 neonates annually. High-risk and critically ill patients represent a significant portion of the admitted neonates. As a level IV NICU, it manages extremely premature neonates and those with complex medical and surgical conditions. It offers various therapeutic modalities such as therapeutic hypothermia, complex neonatal surgeries, and extracorporeal membrane oxygenation. With the high acuity status of the NICU at our center, the employment of neonatal PICC in the management of patients is considerably high. At our institution, a catheter with a small caliber of about 1–2 Fr is typically used in neonates. It is usually inserted by a neonatologist through a superficial vein in the upper or lower extremities. The use of near infrared spectrometer facilitates the visualization of the vein where the neonatal PICC is to be inserted through. Such lines are removed if they are no longer required, and usually the period of use doesn’t exceed 14 days. This study investigates the incidence of life-threatening pericardial and pleural effusions secondary to PICC in neonates in the NICU at the AUBMC. It seeks to characterize the clinical presentation, management, and outcome of neonates who developed tamponade, large pericardial, or pleural effusions secondary to neonatal PICC insertion. It also addresses potential causes of such complications, emphasizes the significance of prompt identification and intervention, and offers suggestions to mitigate this risk.

## 2. Methodology

After securing the institutional review board approval, we performed a retrospective review of charts of neonates who were admitted to the NICU at AUBMC between January 2010 and January 2020, and who required neonatal PICC insertion for various reasons. Neonates who developed tamponade, large pleural or pericardial effusions secondary to PICC insertion were identified. The medical records of these patients including their daily progress notes, echocardiographic findings, chest imaging, laboratory results, and procedural notes were reviewed and analyzed.

## 3. Results

During the period 2010–2020, a total of 810 neonatal PICCs were inserted. Four neonates at our NICU developed large pleural effusions, pericardial effusions, or tamponade that were linked to PICC use, at a yearly incidence rate of 0.049%. Three of the patients were extremely premature neonates of very low birthweight. Remarkably, the four patients were receiving parenteral nutrition through the neonatal PICC. The interval between PICC insertion and clinical deterioration was 1–16 days. All of them experienced sudden deterioration in clinical status, acute cardiopulmonary decompression, and vague non-specific symptoms such as bradycardia, desaturation, and/or hypotension. Two patients required urgent intervention *via* pericardiocentesis. One patient necessitated placement of a chest tube. The pericardial effusions were monitored clinically and through imaging until spontaneous resolution in one patient. No deaths were encountered. Biochemical analysis of the drained fluid was consistent with extravasation of lipid component of TPN. Remarkably, the four patients had correctly positioned PICC line that was confirmed through chest radiography obtained following insertion. The characteristics of the four cases and their complications are summarized in Tables 1, 2. Below is a detailed description of each case.

**TABLE 1:** Characteristics of neonates.

Catheter characteristics	Insertion site	Duration, from insertion to time of event (days)	Age at insertion	CGA at insertion
Case I	Right basilic vein	8 days	3 days	24 weeks + 3 days
Case II	Right basilic vein	4 days	2 days	32 weeks
Case III	Right basilic vein	16 days	24 days	29 weeks
Case IV	Left basilic vein	5 days	12 days	26 weeks + 4 days

**TABLE 2:** Characteristics of complications related to neonatal PICC.

	Clinical presentation	Complication	Diagnostic modality	Management	Outcome
Case I	Hypotension and decrease in oxygen saturation	Pleural effusions, collapse of left lung	Chest X-ray	Chest tube insertion	Resolution of effusions in 2 days
Case II	Hypotension	Pericardial effusions	Chest X-ray Echocardiography	Serial echocardiographic surveillance	Resolution of effusions in 7 days
Case III	Persistent decrease in oxygen saturation	Pericardial effusions, pending tamponade	Chest X-ray Echocardiography	Pericardiocentesis	Resolution of effusions in 2 days
Case IV	Hypotension, bradycardia, decrease in oxygen saturation	Pericardial effusions/tamponade	Echocardiography	Pericardiocentesis	Resolution of effusions in 2 days

### 3.1. Case I

**FIGURE 1:**
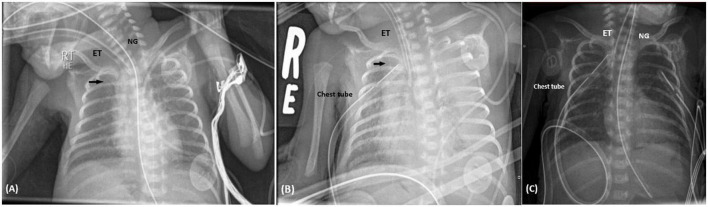
**(A)** Supine chest radiograph following PICC insertion in Case I. Chest X-ray performed in supine position, revealed the PICC inserted through the right basilic vein reaching the left subclavian vein. According to this image, the PICC was retracted 2.5 cm. The black arrow identifies the PICC. NG, nasogastric tube; ET, endotracheal tube. **(B)** Supine chest radiograph following the sudden deterioration in clinical status. Chest X-ray was carried out when the patient had sudden cardiopulmonary decompression. It revealed total whitening of the left lung, as shown on the left side of the image, representing total collapse with left sided pleural effusion. The PICC is represented by the black arrow. ET, endotracheal tube. **(C)** Chest imaging 2 days following chest tube insertion. Chest X-ray showed complete resolution of the pleural effusion, and normal aeration of the left lung. NG, nasogastric tube; ET, endotracheal tube.

A 667-gram newborn female, product of 24-week triplets’ gestation, was delivered *via* cesarean section due to preterm premature rupture of membranes, to a 32-year-old mother. APGAR scores were 7 and 8 at 1 and 5 min, respectively. The newborn was admitted to NICU for the management of extreme prematurity, extreme low birth weight, and respiratory distress. She required intubation and surfactant administration. On the first day of life, umbilical venous and umbilical arterial catheters were placed for blood withdrawal, and for administration of fluids, antibiotics, and nutrition. On the third day of life, PICC was inserted through the right basilic vein for administration of total parenteral nutrition. Chest imaging was performed after insertion and showed the line through the left brachiocephalic vein, with the tip reaching the subclavian vein region. Accordingly, the PICC was retracted 2.5 cm (Figure 1A). Her stay was complicated by right sided pneumothorax on the 11th day of life that required chest tube insertion. On her 16th day of life, she developed sudden decrease in blood pressure reaching a systolic pressure of 35 mmHg, associated with frequent episodes of decreased oxygen saturation down to 60%. As a result, the ventilator settings were adjusted, the peak inspiratory pressure/positive-end expiratory pressure (PIP/PEEP) were increased from 17/3 to 21/5 cm H_2_O, FiO2 was increased, and she was given a bolus of normal saline. Arterial blood gases at that time were significant for acidosis, with a pH of 7.13, pCo2 of 70 mmHg, and base deficit of −7. Urgent imaging of chest was obtained and revealed moderate left-sided pleural effusions with collapse of the left lung (Figure 1B). Point of care ultrasound was performed and confirmed the presence of moderate to large left-sided pleural effusions reaching the apex with underlying collapse of the lung. Interestingly, the pleural effusions appeared slightly complex containing internal debris on ultrasound. Subsequently, left chest tube was inserted, which drained 27 ml of milky white fluid. Thereafter, the PICC line was removed and another one was inserted through the right femoral vein. Biochemical analysis of the fluid was remarkable for elevated triglycerides of 2,427 mg/dL, elevated glucose of 509 mg/dL, proteins of less than 2 g/dL, and albumin of less than 2 g/dL. Blood tests showed plasma triglycerides of 82 mg/dL, protein 45 g/dL, and albumin 33 g/dL. The results were consistent with pleural effusion secondary to TPN extravasation. Chest imaging performed 2 days later showed resolution of the pleural effusions (Figure 1C). In addition, the patient had marked improvement in clinical status.

### 3.2. Case II

A 1,630-gram female, product of 31 weeks 3 days of gestation, was born to a 30-year-old female G3P2L2 *via* urgent C-section due to preterm labor and scar pain. Pregnancy was complicated by prenatal diagnosis of trisomy 13 *via* amniocentesis. APGARs were 5 and 6 at 1 and 5 min, respectively. She was born apneic without spontaneous cry. She required resuscitation and intubation and was admitted to the NICU. Umbilical arterial and umbilical venous catheters were inserted on the first day of life. Echocardiography was performed which revealed large patent ductus arteriosus (PDA), patent foramen ovale, atrial septal defect and mild tricuspid regurgitation. Therefore, she was restricted in fluid intake and started on Paracetamol. Right upper extremity PICC was inserted through the basilic vein on the second day of life, through which she was started on TPN. The next day, the patient was acidotic of pH 7.2, with frequent episodes of decrease in oxygen saturation. She had increasing pressure and oxygen requirements. Chest imaging showed ground glass opacities with air bronchogram keeping with hyaline membrane disease. The patient had continuous clinical deterioration; therefore, she was switched to high frequency oscillator. On the fourth day of life, follow up echocardiography was significant for a decrease in the PDA size; however, PICC was visualized in the right atrium. Based on the imaging, PICC was retracted to the proper position. Few hours later, she developed hypotension reaching 36/16 mmHg, for which two normal saline boluses were administered with improvement in blood pressure. Bedside echocardiography revealed moderate pericardial effusions. The decision was taken to monitor the patient clinically for any signs of deterioration such as desaturation, bradycardia, or hypotension that would trigger prompt intervention. Addition, daily echocardiographic follow up was planned to monitor the size of the pericardial effusion. Serial echocardiographic examination showed progressive decrease and spontaneous resolution of effusions.

### 3.3. Case III

**FIGURE 2:**
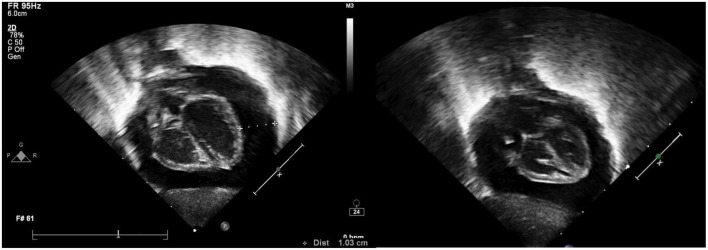
Echocardiogram showing cardiac tamponade of Case III.

A 730-gram male newborn, product of 25 weeks and 4 days of gestation, member of twin, conceived *via in vitro* fertilization, was delivered by C-section for suspected placental abruption, to a 34-year-old female. The pregnancy was complicated by oligohydramnios, multiple episodes of vaginal bleeding that required hospitalization, and suspected prolonged rupture of membranes. Notably, the mother received full course of steroids at 24 weeks of gestation, and ampicillin and clarithromycin for suspected membrane rupture. APGARs were 6 and 8 at 1 and 5 min, respectively. On admission, umbilical arterial and umbilical venous catheters were inserted. A PICC was inserted on the 10th day of life through the left upper extremity and removed at 2 weeks from insertion. Another PICC was inserted through the right basilic vein on the 24th day of life. During his hospital stay, he developed right sided pneumothorax that required needle decompression and chest tube insertion and failed three attempts of removing mechanical ventilation. On the day of life 40, the patient started having repetitive episodes of decrease in oxygen saturation down to 58%. Chest imaging revealed diffuse granular infiltrates. Echocardiography showed large pericardial effusions, in pending tamponade (Figure 2). In addition, the tip of the central venous catheter was seen in the right atrium. The PICC was withdrawn 2 cm and urgent pericardiocentesis was performed under echocardiographic guidance. The patient had hypotension during the procedure reaching 30/20 mmHg that responded to fluid bolus. During the procedure, 25 ml of clear fluid was drained. Studies taken from tapped fluid remained negative for infectious workup. Nevertheless, biochemical analysis revealed the presence of protein of 1.9 g/dL, albumin of <1.7 g/dL, LDH of 34 U/L, and triglycerides of 47 mg/dL. At that time, serum protein was 46 g/dL, serum albumin of 26 g/dL, serum triglycerides of 186 mg/dL, and serum LDH 243 U/L. Follow up echocardiography which was performed 2 days after the pericardiocentesis showed good biventricular function without evidence of pericardial effusions.

### 3.4. Case IV

A 540-gram female neonate, product of 24 weeks and 5 days of gestation, was born *via* C-section, due to maternal pre-eclampsia with severe features, and suspected placenta abruption. She was transferred to our institution at the seventh day of life for further management of extreme prematurity, extreme low birth weight and respiratory distress syndrome. On admission, the patient had umbilical arterial and venous catheters in place. Additionally, a PICC was inserted through the left upper extremity for administration of medications and TPN. The position of the catheter was confirmed *via* chest X-ray. On the 12th day of life, the patient had acute onset hypotension, associated with bradycardia down to 70 beats/min and decrease in oxygen saturation. Point of care echocardiography showed large pericardial effusion/tamponade. Urgent bedside pericardiocentesis was performed that yielded milky fluid. After pericardiocentesis, the patient hemodynamic status significantly improved. Follow-up imaging with echocardiogram revealed resolution of the effusion with good biventricular systolic function, at 2 days following the procedure.

## 4. Discussion

The four cases described in our study represent a rare yet critical complication of neonatal PICCs. All patients exhibited sudden hemodynamic instability and required prompt intervention. With the tremendous increase in survival rate of extremely premature and critically ill neonates, the use of neonatal PICCs has been on rise and is now considered essential in supporting these neonates. Data regarding neonatal PICC-associated pericardial effusion/tamponade originated initially from case reports described by neonatologists around the world (7, 8). Only recently large retrospective studies tackling neonatal PICC-related complications have shed the light on this serious problem. The estimated incidence of pericardial effusions secondary to central venous catheter varies among the different studies but remains at a low level. However, the estimated mortality rate is up to 50% (9). In a nationwide Japanese survey, the incidence of pericardial effusions/tamponade ranged between 0.07 and 0.11% (10), keeping with the results of another nationwide survey performed in the United Kingdom that revealed estimated incidence of 0.18% (9). Another two large retrospective studies in Australia and Brazil reported incidence rate of 0.05 and 1.5%, respectively (11, 12). Interestingly, Nadroo et al. surveyed 82 NICUs in the United States and found that 43 and 20% experienced the incidence of at least one pericardial effusion and at least one death due to tamponade secondary to neonatal PICC, respectively (8). As a result, even though there is a consensus among various studies regarding the rarity of pericardial effusions complicating PICC insertion, a significant proportion of NICUs still experience it. Similarly, pleural effusions might complicate up to 2.2% of PICCs inserted in the NICUs (11, 13). In our study, three out of the four cases were extremely premature neonates, with extremely low birth weight. Nevertheless, studies have not discovered a correlation between gestational age or weight and the incidence of tamponade secondary to neonatal PICC (14–16). The management of pericardial effusions is controversial and greatly depends on the size of the effusions and the patient’s clinical status. Based on simple semiquantitative echocardiographic evaluation, pericardial effusions can be classified into mild (less than 10 mm), moderate (10–20 mm), and large/severe effusions (>20 mm) (17). In fact, small pericardial effusions, unlike moderate and severe cases, are often asymptomatic, detected incidentally, and require only clinical surveillance (17). On the other hand, the clinical presentation and the patient’s hemodynamics substantially hang on the velocity of fluid accumulation and influence the decision to intervene. When the pericardial effusion accumulates rapidly, or presents in a tamponade, pending tamponade, or is causing significant respiratory or hemodynamic instability, pericardiocentesis is usually performed (18, 19).

**FIGURE 3:**
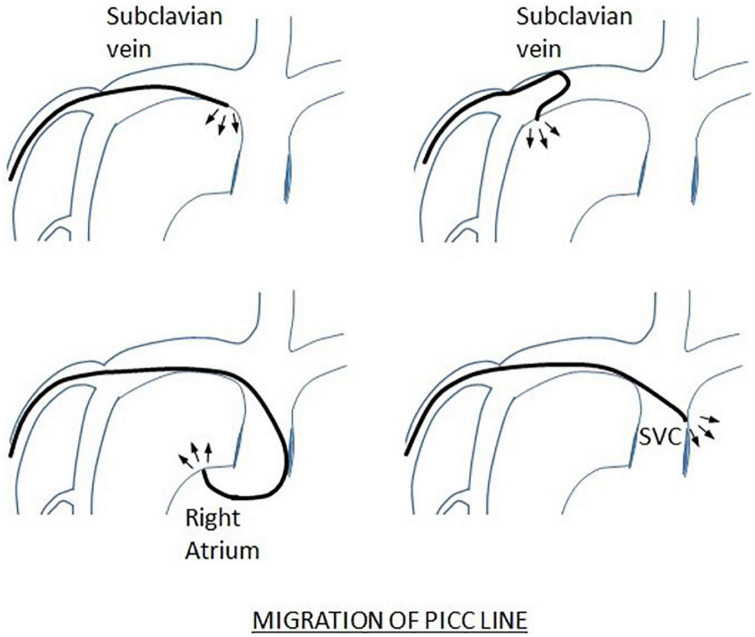
Peripherally inserted central catheters migration leading to vascular perforation. One of the potential etiologies behind pericardial and pleural effusions is chronic friction resulting in vascular wall compromise and ultimate perforation. As the line migrates, the catheter tip might meet the continuously moving vessel wall. Subclavian vein, superior vena cava and the right atrium are potential sites of injury. The thin vascular walls, and the rapid heart rate contribute to increased frequency and impact of friction. The angle formed by the line and vascular wall is important. When the angle is relatively perpendicular, direct vessel trauma and erosion occur, leading to thrombus formation and further catheter adherence to the vascular structure. Eventually, erosion is potentiated, which might lead to preformation and fluid accumulation in the pericardium (35).

**FIGURE 4:**
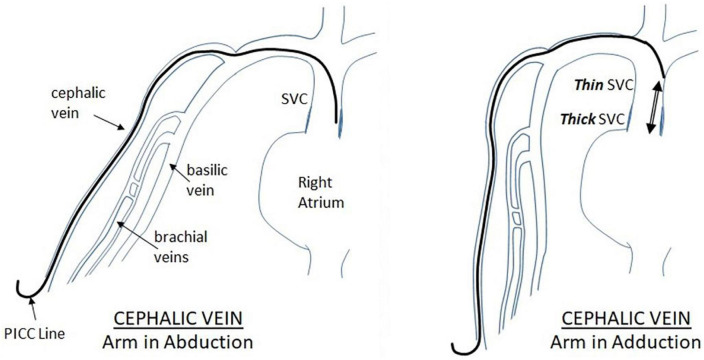
Displacement of PICC inserted through the cephalic vein during arm movement. As illustrated in the figure, when the upper extremity is moving from abduction to adduction position, the PICC inserted *via* the cephalic vein moves in a cephalic fashion, away from the heart.

**FIGURE 5:**
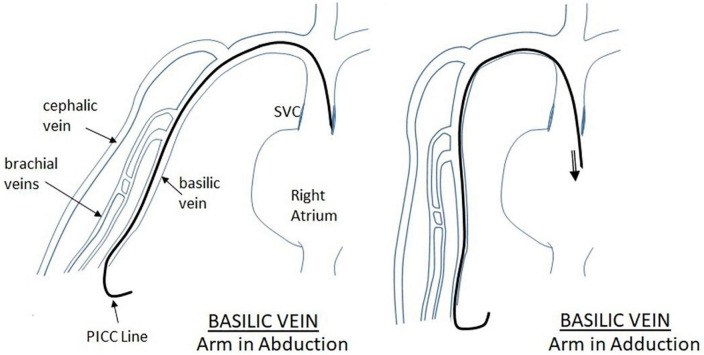
Displacement of PICC inserted through the Basilic vein during arm movement. When the arm is moved from abduction position to adduction, there is tendency for the PICC to move inward toward the heart. This should be considered during the final PICC placement.

Such a devastating complication is multi-factorial in origin. Overall, it can be caused by mechanical or chemical insult. Mechanically, the catheter tip positioning is a crucial player in development of effusions. As the tip of the catheter is inserted, it might meet the cardiac or vascular wall resulting in chronic friction and erosion. Because newborns have a small right atrium, a thin atrial and vascular wall, and a rapid heart rate, continuous mechanical stimulation of the catheter tip may rupture the atrium leading to a sub-acute pericardial effusions (20). The presence of the catheter tip in the right atrium on imaging is more commonly associated with this complication (12). In a more acute setting, it can be caused by direct cardiac perforation during insertion (21). Even with appropriate positioning of the catheter, line migration might occur. Figure 3 illustrates the possible scenarios regarding PICC migration leading to vascular wall erosion. Indeed, about 24% of PICCs migrate within the first 24 h post-insertion (16). Migration is more likely to occur when the line is placed through the upper extremities (16). Arm adduction might trigger the tip of the catheter to move an average of 2–3.3 cm (22). During adduction of the upper extremity, as illustrated in Figures 4, 5, PICCs inserted through the basilic vein move toward the heart while those inserted *via* the cephalic vein move away, while flexion of the elbow causes both lines to move toward the heart (23). Displacement of catheter can also be triggered by neck position, frequent flushing of the central line, loosening of the dressing, manipulation of the PICC during dressing change, and growth of the neonate (24). Another potential mechanism of vascular erosion is related to the hyperosmolarity of the TPN components, especially fat emulsions. It might penetrate through the right atrial wall, without obvious myocardial damage, leaving behind interstitial edema and dilatation of the fine vascular channels within the epicardial soft tissue (25). Ultimately, endocardial cell damage takes place. In all cases, the result is fluid accumulation in the pericardial cavity. Subsequently, the pericardial pressure increases reaching a critical level at which the cardiac output is compromised, resulting in cardiac shock. Indeed, two of the presented cases had effusions secondary to TPN extravasation. Similarly, a study by Barbosa et al. at a level III NICU, revealed that 20% of cases were associated with TPN (26).

To diminish the threat of such disastrous complication and its severe outcomes, appropriate catheter placement, frequent surveillance, low clinical suspicion, rapid diagnosis, and timely intervention are necessary. Importantly, any central venous catheter should be removed in the absence of judicious indication for placement. Whereas the ideal PICC position, when needed, is controversial, the tip of the PICC should be located outside the pericardial reflection line, meaning at a level above T2 on the chest radiograph. This was supported by Nowlen in his case series, where the catheter tip was last reported within the pericardial reflection in 92% of cases (27). Routine chest imaging following neonatal PICC insertion should be obtained to identify the location of the tip. It needs to be visualized at the intersection of the superior vena cava and right atrium. Catheter should be regularly checked for tip migration through chest imaging. Some studies suggest the use of ultrasound to be superior to X-rays in routine inspection of catheter location (28). It is also suggested that the use of real-time ultrasound during catheter insertion is associated with significantly reduced risk of tip mal positioning (29). Some institutions perform routine chest imaging twice weekly to monitor the position of the catheter tip (30). Besides, proper dressing has a role in reducing dislocation. Therefore, institutional policy should include frequent surveillance of catheter dressing. The use of medical cyanoacrylate glue is proposed to be superior to conventional dressing in reducing the incidence of catheter dislocation (31). Also, we suggest that at any time, should a chest radiograph be performed for any clinical purpose, the neonatal PICC tip must be reviewed. However, chest imaging alone to check appropriate catheter position remains suboptimal. Therefore, at the slightest suspicion, prompt evaluation with ultrasound must be performed to save valuable time and prevent catastrophic events. Point of care ultrasound has proven to be a reliable and effective modality to accurately determine the position of central catheters, and to evaluate for potential complications (32, 33). Even the role of echocardiographic assessment has changed significantly over the past years. While it was exclusively performed by a pediatric cardiologist, neonatologists have become increasingly interested by point of care echocardiography to evaluate cardiac function, in the setting of acute clinical instability in patients without previous evidence of congenital heart disease (34). Finally, repeating echocardiographic evaluation is recommended in case of any further deterioration in patient’s clinical status.

## 5. Conclusion

This study emphasizes the potential risk of developing massive pleural, pericardial effusions, or cardiac tamponade with neonatal PICC use at the NICU. Proper catheter position and routine position checking are highly important to reduce the risk of developing life-threatening effusions. In patients with acute hemodynamic instability, low threshold to suspect PICC related effusions is crucial to avoid serious consequences. Bedside transthoracic echocardiography enables prompt identification of effusions or tamponade. The emergent use of pericardiocentesis provides rapid hemodynamic improvement in most patients.

## Data availability statement

The raw data supporting the conclusions of this article will be made available by the authors, without undue reservation.

## Ethics statement

The studies involving human participants were reviewed and approved by the Institutional Review Board at the American University of Beirut Medical Center. Written informed consent from the participants’ legal guardian/next of kin was not required to participate in this study in accordance with the national legislation and the institutional requirements.

## Author contributions

MAr conceived the presented idea and the study framework. RZ, MAn, and TH performed the data collection, analysis, and wrote the first draft of the manuscript. IE helped in the analysis and construction of figures. FB, MAr, and KY supervised the project and did the final editing. All authors contributed to corrections and adjustment of subsequent iterations of the manuscript, and approve and agree with the content.
